# Improved SSVEP Classification Through EEG Artifact Reduction Using Auxiliary Sensors

**DOI:** 10.3390/s26030917

**Published:** 2026-01-31

**Authors:** Marcin Kołodziej, Andrzej Majkowski, Przemysław Wiszniewski

**Affiliations:** Faculty of Electrical Engineering, Warsaw University of Technology, Pl. Politechniki 1, 00-661 Warsaw, Poland; marcin.kolodziej@pw.edu.pl (M.K.); przemyslaw.wiszniewski.dokt@pw.edu.pl (P.W.)

**Keywords:** EEG, EMG, EOG, BCI, artifact reduction, artifact elimination, SSVEP, auxiliary sensors

## Abstract

Steady-state visual evoked potentials (SSVEPs) are one of the key paradigms used in brain–computer interface (BCI) systems. Their performance, however, is substantially degraded by EEG artifacts of muscular, motion-related, and ocular origin. This issue is particularly pronounced in individuals exhibiting increased facial muscle tension or involuntary eye movements. The aim of this study was to develop and evaluate an EEG artifact reduction method based on auxiliary channels, including central (Cz), frontal (Fp1), electrooculographic (HEOG), and muscular electrodes (neck, cheek, jaw). Signals from these channels were used to model the physical sources of interference recorded concurrently with occipital brain activity (O1, O2, Oz). EEG signal cleaning was performed using linear regression in 1-s windows, followed by frequency-domain analysis to extract features related to stimulation frequencies and SSVEP classification using SVM and CNN algorithms. The experiment involved three visual stimulation frequencies (7, 8, and 9 Hz) generated by LEDs and the recording of controlled facial and jaw-related artifacts. Experiments conducted on 12 participants demonstrated a 9% increase in classification accuracy after artifact removal. Further analysis indicated that the Cz and jaw channels contributed most significantly to effective artifact suppression. The results confirm that the use of auxiliary channels substantially improves EEG signal quality and enhances the reliability of BCI systems under real-world conditions.

## 1. Introduction

Brain–computer interfaces (BCIs) enable direct communication between neural activity and external systems without engaging conventional motor pathways [1,2]. Among noninvasive BCI modalities, steady-state visual evoked potentials (SSVEPs) recorded over occipital areas are widely used due to their high signal-to-noise ratio, simple hardware requirements, and capacity to encode multiple commands via distinct stimulation frequencies [1,3]. These properties have led to applications in assistive communication, device control, and augmented reality [3]. A major limitation of SSVEP-based BCIs is their high sensitivity to non-cerebral artifacts, including electromyographic (EMG) activity from facial, jaw, and neck muscles, electrooculographic (EOG) artifacts related to eye movements and blinking, and motion-induced disturbances [4,5]. While such artifacts can be partially controlled in laboratory settings, real-world usage inevitably involves head movements, facial expressions, and speech, which substantially degrade SSVEP signal quality and classification performance [4,6]. Effective artifact elimination without constraining natural user behavior is therefore a key challenge in BCI design. Existing EEG artifact reduction methods—such as ICA, PCA-based techniques, adaptive filtering, and deep learning approaches—can be effective but often require high computational cost, extensive parameter tuning, or manual component selection, limiting their suitability for real-time and mobile BCI systems [5,7]. An increasingly promising approach is the use of auxiliary channels placed in regions prone to artifact generation, including frontal and periocular sites, facial muscles, and the neck [4,5]. These channels are not intended to capture cortical SSVEP activity directly, but rather to model artifact sources and their projection onto occipital electrodes, enabling physiologically interpretable artifact suppression through regression-based methods [4].

Although SSVEP research has largely focused on advanced classification techniques such as CCA, TRCA, and deep neural networks [8,9], their effectiveness is fundamentally limited when artifact contamination is not adequately addressed at the signal level [5]. This suggests that relatively simple artifact correction strategies based on well-chosen auxiliary channels may yield gains comparable to, or greater than, increasing classifier complexity alone [10]. Despite growing interest in auxiliary electrodes, systematic analyses of their individual contributions—particularly muscular channels placed on the cheeks, jaw, or neck—remain scarce. Moreover, many studies rely on experimental protocols that suppress natural movements, limiting ecological validity [11]. Deliberately eliciting facial and muscular activity allows quantitative assessment of artifact impact and objective evaluation of artifact reduction methods using SSVEP performance metrics such as accuracy and information transfer rate (ITR) [5].

In this study, we propose a linear regression–based artifact reduction method for SSVEP BCI using auxiliary channels including Cz, Fp1, HEOG, and muscular electrodes on the neck, cheek, and jaw. These signals are used to estimate and subtract artifact components from occipital electrodes (O1, O2, Oz), yielding a cleaned occipital signal. Performance is evaluated by comparing conventional occipital-only analysis with the artifact-cleaned approach using identical feature extraction (FFT) and two classifiers (linear SVM and compact CNN). A key contribution is a systematic analysis of the relevance of individual auxiliary channels, addressing their necessity, redundancy, and consistency across participants. Identifying a minimal yet effective set of auxiliary electrodes is crucial for practical BCI deployment, as each additional channel increases system complexity, preparation time, and cost. The presented approach supports informed trade-offs between simplicity and robustness, guiding the design of SSVEP-based BCI intended for reliable real-world operation [12].

## 2. Related Articles

Steady-state visual evoked potentials (SSVEPs) are among the most established EEG signals used in BCI systems due to the high repeatability of visual cortex responses and the ability to encode multiple commands via stimulus frequency modulation [13,14,15]. In practice, system performance depends not only on stimulus design but also on the robustness of signal processing methods to noise and inter-subject variability [14,16]. Classical SSVEP recognition relies on correlation-based methods, most notably canonical correlation analysis (CCA), which maximizes correlation between multichannel EEG and sinusoidal reference signals [17]. Extensions such as filter bank CCA (FBCCA) improve performance by exploiting sub-band and harmonic information [18], while synchronization-based methods like MSI show advantages for short time windows and limited channel counts [19]. Data-driven spatial filtering approaches, particularly task-related component analysis (TRCA) and its variants, further enhance signal-to-noise ratio and often outperform CCA in multi-target and short-latency scenarios [9,20,21]. Hybrid spatial–spectral and TRCA–CCA approaches have also been proposed [22,23,24].

In parallel, deep learning methods have been increasingly explored, shifting from handcrafted features toward raw-signal and time–frequency representations. Recent work includes transformer-based inter-subject SSVEP classifiers and comprehensive surveys of CNN-, RNN-, and transformer-based approaches [25,26]. These developments are accompanied by growing interest in few-channel and wearable systems, where signal-to-noise degradation and artifact contamination are particularly pronounced [27,28]. Across methods, stimulus properties and user comfort remain critical factors influencing SSVEP amplitude and long-term stability [29,30].

Despite methodological advances, most SSVEP-based BCIs are evaluated under laboratory conditions that minimize movement and facial muscle activity. Robustness studies demonstrate that motion, muscle activation, and electrode–skin changes can markedly degrade SSVEP performance, with muscle artifacts identified as a key limiting factor, especially in mobile scenarios [31,32]. Consequently, there is increasing demand for artifact elimination methods that are computationally lightweight, suitable for online use, and physiologically interpretable. Reviews of EEG artifact correction methods distinguish regression-based, adaptive, decomposition-based (ICA/PCA/CCA), subspace, and machine-learning approaches, highlighting the trade-off between artifact suppression and neural signal distortion [4,5,33,34]. In SSVEP systems, this issue is particularly critical, as residual EMG contamination within harmonic bands directly affects spectral features used for classification, especially for short decision windows. Linear regression methods, including least-squares (LS), are among the earliest and most established artifact removal techniques, particularly for ocular artifacts [35,36,37]. The Gratton–Coles–Donchin procedure (EMCP) models artifact propagation from reference channels and subtracts the estimated contribution from EEG signals [35]. Regression-based approaches are valued for their simplicity, low computational cost, and suitability for real-time applications, although their limitations—such as mutual EEG–EOG contamination and temporal variability—are well documented [37,38]. Adaptive extensions (e.g., LMS, RLS) address nonstationarity caused by posture changes, muscle tension, and movement, forming a coherent family of methods with increasing flexibility at the cost of higher complexity [39,40,41]. Compared with regression, ICA-based methods can effectively separate artifact components but require more channels, careful component selection, and are sensitive to implementation details [37,42]. Their widespread adoption was facilitated by tools such as EEGLAB [7], and seminal studies demonstrated successful removal of EOG and EMG artifacts with appropriate reconstruction strategies [42]. Subspace-based techniques offer intermediate solutions between regression and full ICA decomposition [43].

Muscle artifacts pose a particular challenge for SSVEP systems due to their broadband, often nonlinear nature and strong projection onto occipital electrodes, where they can mask SSVEP harmonics. Dedicated EMG reduction methods, including multi-stage and assisted approaches using auxiliary EMG references, have shown that physically grounded channels can improve denoising effectiveness compared with purely statistical techniques [44,45,46]. From an SSVEP perspective, direct comparisons of artifact removal methods indicate that performance gains strongly depend on artifact type and reference channel selection [10]. Overall, the literature emphasizes the growing importance of artifact-robust BCI under naturalistic conditions involving movement and facial activity [15,31,34]. While recent trends focus on increasingly complex classifiers [9,20,21,25,26], signal quality remains a fundamental bottleneck in the presence of EMG and EOG artifacts. In this context, LS regression using auxiliary artifact-recording channels represents an attractive engineering solution: it is computationally efficient, suitable for online implementation, and provides interpretable coefficients quantifying the contribution of individual artifact sources [10,35,39]. This approach aligns with current trends toward assisted denoising using additional sensors [45] and the optimization of minimal electrode sets for wearable and low-density SSVEP-based BCIs [28,47].

## 3. Aim of the Article

**Figure 1:**
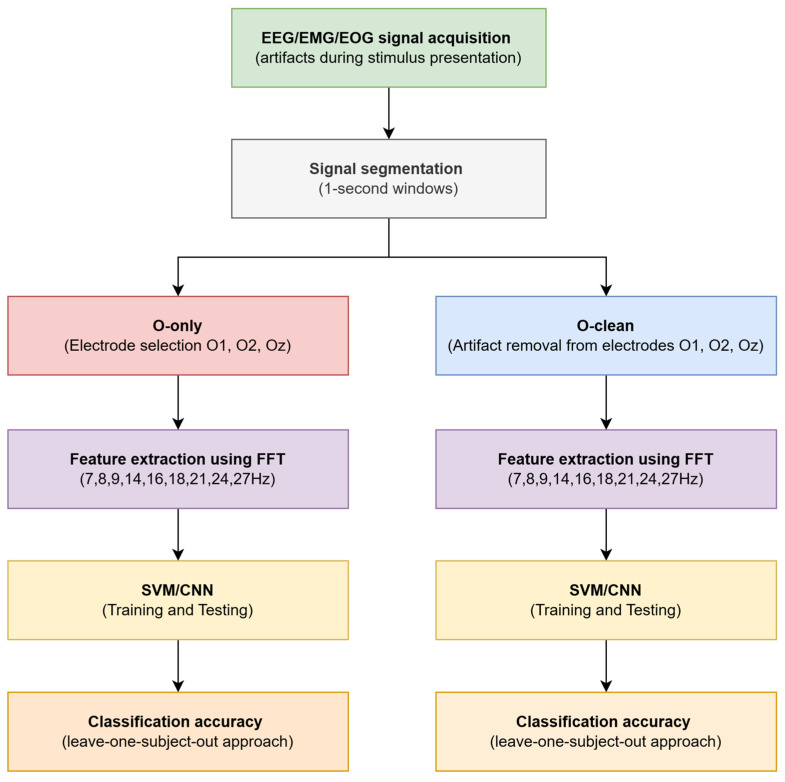
Block diagram of the experimental procedure applied in the analysis of EEG/EMG/EOG signals.

The aim of this study is to develop and compare the effectiveness of an EEG artifact reduction method for SSVEP-based BCI systems using different configurations of auxiliary channels. The analysis focuses on identifying which additional channels—covering central, frontal, electrooculographic, and muscular electrodes—contribute most to the suppression of non-cerebral interference. Understanding these relationships is essential for optimizing the number and placement of electrodes in practical BCI systems, thereby improving classification accuracy and operational reliability under real-world conditions. Figure 1 presents a block diagram of the EEG/EMG/EOG signal processing procedure applied in this study.

The diagram illustrates the conceptual workflow comprising two parallel EEG processing pipelines: O-only and O-clean. In the first stage, EEG, EMG, and EOG signals were recorded during visual stimulus presentation. The collected data were then segmented into 1-s epochs to ensure uniform temporal analysis windows. In the O-only pipeline, only occipital electrodes (O1, O2, Oz) were used, representing the conventional approach in SSVEP analysis. In contrast, the O-clean pipeline additionally applied artifact suppression to signals from the same occipital electrodes using linear regression with auxiliary channels to model non-cerebral interference.

In both pipelines, feature extraction was performed using the fast Fourier transform (FFT), analyzing amplitudes in frequency bands corresponding to the stimulation frequencies and their harmonics (7, 8, 9, 14, 16, 18, 21, 24, and 27 Hz). The extracted features were subsequently used for classification with SVM and CNN algorithms, employing a leave-one-subject-out approach to assess generalized performance. The study design is based on a direct comparison of classification results obtained from the O-only and O-clean pipelines, enabling quantitative assessment of the impact of artifact reduction on SSVEP recognition performance. In addition, the analysis includes a search for optimal combinations of auxiliary channels that yield the greatest improvement in classification accuracy, allowing recommendations for a minimal yet effective electrode set for practical BCI applications. In summary, the objectives of this work are to:

- Develop and experimentally validate an EEG artifact reduction method using auxiliary channels in an SSVEP–BCI system;
- Quantitatively evaluate the impact of signal cleaning on classification accuracy and information transfer rate using SVM and CNN models;
- Identify the auxiliary channels that contribute most to signal quality improvement and may form the basis of a minimal yet effective electrode configuration for practical applications.

The results demonstrate that even a relatively simple linear regression approach, when combined with a physiologically motivated arrangement of auxiliary channels, can lead to a substantial improvement in the reliability of SSVEP-based BCI systems under conditions close to real-world use.

## 4. Materials

**Figure 2:**
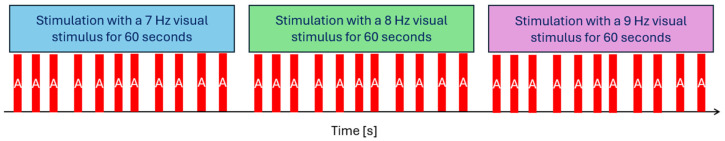
Temporal scheme of the visual stimulation protocol used in the study. Three consecutive stages of visual stimulation at frequencies of 7 Hz, 8 Hz, and 9 Hz are shown, each lasting 60 s. Artifact episodes are marked in red and labeled with the letter A along the timeline.

To record EEG signals containing SSVEP responses contaminated by muscle artifacts, an experimental protocol was employed in which participants observed visual stimuli at frequencies of 7, 8, and 9 Hz while simultaneously generating muscle-related artifacts. Each participant completed three recording sessions, with approximately one minute of rest between sessions. The entire experiment consisted of three consecutive stages of SSVEP visual stimulation with a total duration of 3 min. In each session (60 s), flickering visual stimulation was delivered using an LED source at a specific frequency: 7 Hz in the first session, 8 Hz in the second, and 9 Hz in the third. The stimulation was provided by a 6 × 6 cm LED panel flickering synchronously at the designated frequencies. The emitted light was green. During each session, participants intentionally performed muscle and facial activities, selected by the user, designed to simulate typical artifacts commonly observed in EEG recordings. Figure 2 illustrates the experimental timeline, showing EEG recording during three consecutive stages of SSVEP visual stimulation.

During each stage, the participant intentionally performed muscular and facial activities designed to emulate typical artifacts encountered in EEG recordings. These episodes included

- Raising the eyebrows and contracting the frontal muscles;
- Jaw clenching and mandibular movements;
- Activation of the neck and cheek muscles;
- Smiling, grimacing, or brief facial expressions.

Artifact episodes (marked in red along the timeline) occurred at random times during stimulation, typically lasting 1–2 s, and were interspersed with periods of stable, artifact-free EEG recording. This experimental design enables analysis of the impact of different types of muscular activity on SSVEP signals and allows for evaluation of the effectiveness of artifact compensation methods.

**Figure 3:**
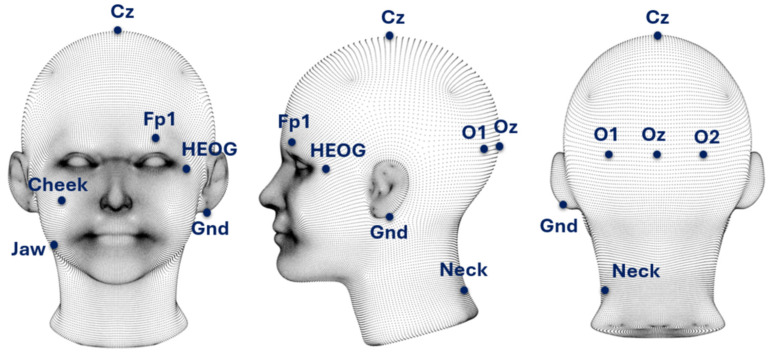
Scheme of EEG and EMG electrode placement used during EEG/EMG/EOG signal acquisition.

The electrodes used for signal acquisition were selected based on previous studies that identified configurations enabling effective simultaneous recording of EEG activity and muscle artifacts [48]. The experimental setup included occipital EEG electrodes O1, O2, and Oz for recording SSVEP responses; EEG electrode Cz for monitoring central cortical activity; EEG electrode Fp1 for monitoring frontal cortical activity; a dedicated HEOG electrode for recording horizontal eye movements; and EMG electrodes placed on the neck, cheek, and jaw for muscle activity recording. In total, nine recording electrodes were used, while the reference electrode was placed on the participant’s earlobe. A schematic diagram of the EEG and EMG electrode placement employed during signal acquisition is shown in Figure 3.

The EEG electrode configuration (O1, O2, Oz, Cz, and Fp1) was designed to ensure high-quality recording of SSVEP responses while simultaneously reducing setup complexity and participant discomfort. Given the visually driven nature of the study, particular emphasis was placed on occipital electrodes located over the visual cortex, where SSVEP signals exhibit the highest amplitude and stability. The Cz electrode enabled observation of the propagation of muscle artifacts into central cortical areas, whereas the Fp1 electrode allowed for monitoring basic oculographic artifacts, such as eye blinks and eye movements. The adopted electrode layout provided sufficient spatial resolution for the intended analyses while shortening preparation time, which was especially important when working with multiple participants. The setup was complemented by EMG electrodes placed on the face and neck (HEOG, neck, cheek, and jaw), enabling precise recording of muscle activity and assessment of the spatial distribution of non-neuronal interference. Signal acquisition was performed using a g.Tec g.USBamp 2.0 bioelectric amplifier, an EEG cap, and g.LADYBIRD active electrodes (g.Tec, Gratz, Austria). Signals were sampled at a frequency of 256 Hz.

Twelve volunteers aged between 18 and 52 years (labeled S01–S12) participated in the study. Each participant was thoroughly informed about the experimental procedure both in written and oral form. After becoming familiar with the protocol, all participants provided voluntary informed consent to take part in the experiment, confirmed by their signature. Prior to recording, participants were fitted with an EEG cap containing active electrodes (O1, O2, Oz, Cz, and Fp1), and EMG electrodes were attached to the face and neck (HEOG, neck, cheek, and jaw) using adhesive patches. Conductive gel was applied to ensure low electrode–skin impedance and stable signal quality.

**Figure 4:**
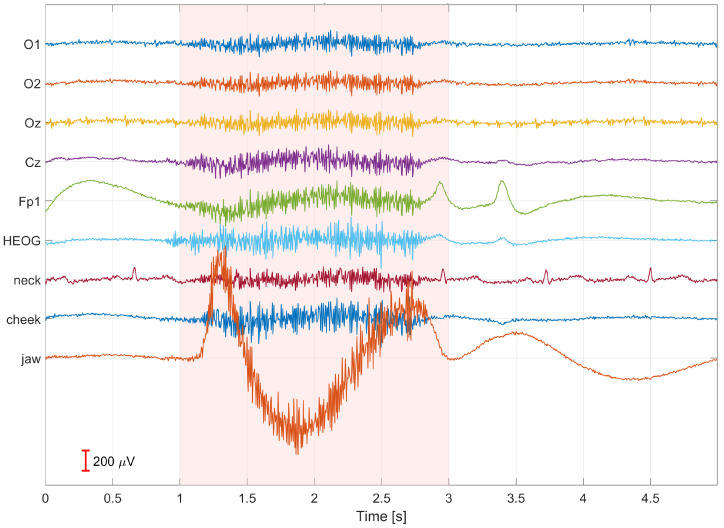
Example of a 5-s segment of signals recorded from participant S01 during 9 Hz visual stimulation while performing artifacts; artifact occurrences are marked in red.

Signal acquisition, processing, and visualization were carried out using custom software developed in the MATLAB R2025b, MathWorks environment. To improve data quality, signal filtering was applied. Power-line interference was removed using a fourth-order Butterworth notch filter with a stopband of 48–52 Hz, while an eighth-order Butterworth band-pass filter in the range of 0.5–100 Hz was used to preserve relevant EEG signal components. No additional signal processing or correction methods were applied during the recording stage. Figure 4 presents an example of a 5-s segment of signals recorded from participant S01 during 9 Hz visual stimulation while intentionally performing artifacts. The figure shows the time courses of bioelectrical signals recorded under conditions of motion- and muscle-related interference. Artifact occurrences are marked in red, allowing for unambiguous identification of their temporal locations and assessment of their impact on the analyzed signal. The recorded EEG signals are publicly available in the database A Database of EEG and EMG SSVEP Recordings for Artifact Analysis and Removal (https://github.com/kolodzima/EEG_artefact_SSVEP_EMG_EOG, accessed on 29 December 2025).

## 5. Methods

### 5.1. EEG Artifact Removal Method

To reduce muscular, motion-related, and ocular artifacts in EEG signals, a linear regression model based on the least squares (LS) method was employed [5,49,50,51,52]. This approach models the artifact component present in occipital EEG signals (O1, O2, Oz) as a linear combination of signals acquired from auxiliary channels (Aux), including central, frontal, oculomotor, and muscular electrodes (Cz, Fp1, HEOG, neck, cheek, jaw). For each 1-s recording window, the regression coefficient vector ***β*** was estimated according to the following model [35,53]:

(1) y=Xβ+ε

where ***y*** denotes the vector of EEG samples from a given occipital electrode, ***X*** is the observation matrix formed from the auxiliary channel signals (augmented with a constant bias term), ***β*** is the vector of regression coefficients, and ***ε*** represents the residual component remaining after artifact removal. The coefficients ***β*** were estimated using the least squares criterion by minimizing the squared error norm ${\left\|\varepsilon \right\|}^{2}$, yielding [54]:

(2) $$\beta ={\left(X^{T}X\right)}^{-1}X^{T}y$$

The cleaned EEG signal was then obtained by subtracting the estimated artifact component from the original signal [54]:

(3) $$y_{clean}=y-X\beta$$

The resulting signal $y_{clean}$ represents an approximation of EEG activity with artifact-related components attenuated. The use of LS regression was motivated by several practical and methodological considerations. First, it is a mathematically simple and well-defined method that does not require hyperparameter tuning or iterative training, making it particularly suitable for large EEG datasets. Its linear nature allows for direct interpretation of the regression coefficients ***β*** as quantitative measures of the contribution of individual auxiliary channels to the artifact model.

**Figure 5:**
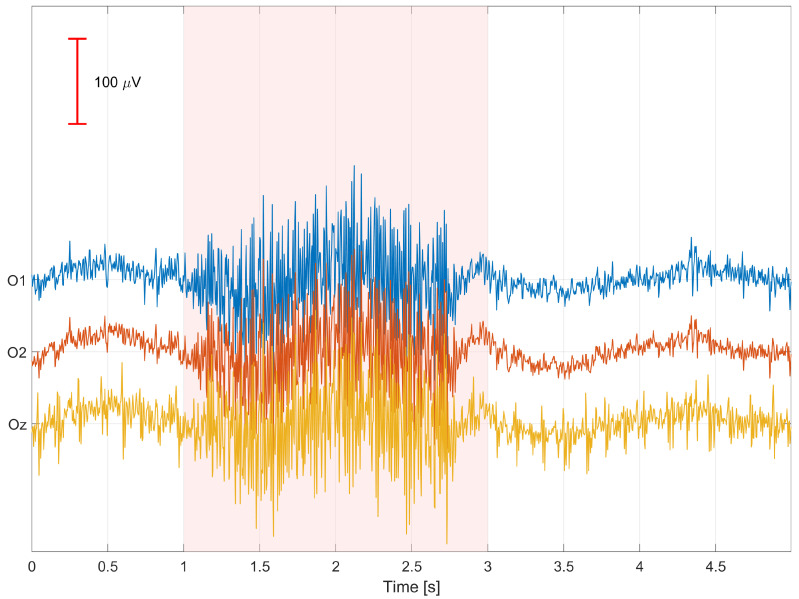
Example of a 5-s segment of signals recorded from the occipital electrodes O1, O2, and Oz, denoted as O-only (before signal cleaning), obtained from participant S01 during 9 Hz visual stimulation; artifact occurrences are marked in red.

**Figure 6:**
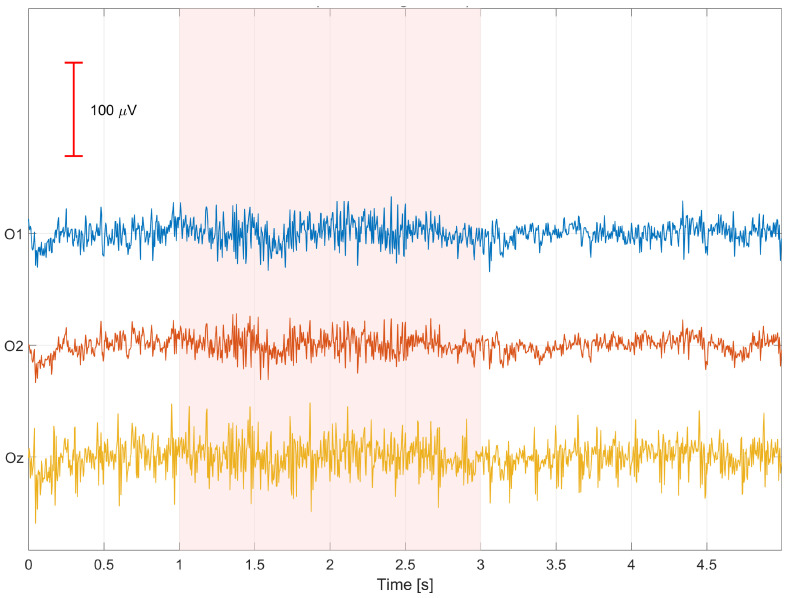
Example of a 5-s segment of signals recorded from the occipital electrodes O1, O2, and Oz after artifact removal (O-clean) using auxiliary channels, obtained from participant S01 during 9 Hz visual stimulation.

**Figure 7:**
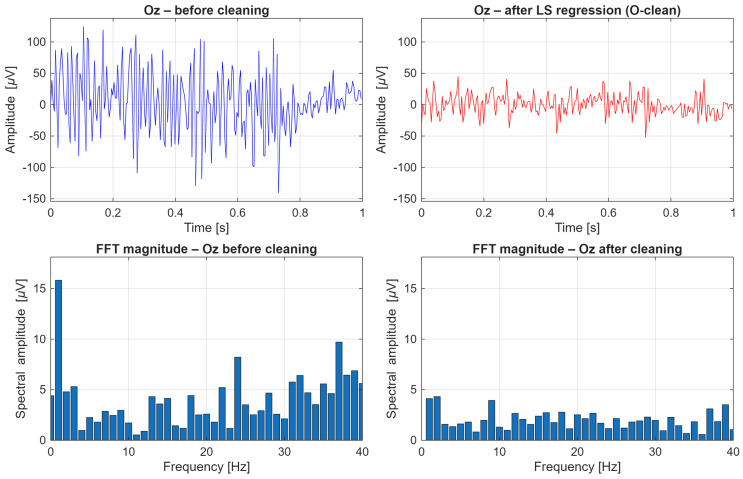
Example of a 1-s EEG window from the Oz electrode in participant S03 during 9 Hz visual stimulation: time-domain waveform and FFT amplitude spectrum before cleaning (O-only) and after artifact removal using least squares regression (O-clean).

Unlike more complex approaches such as ICA, PCA, or spatial source–based methods (e.g., SSP, ASR), LS regression enables explicit modeling of physiologically related channels and their use as regressors with clear anatomical interpretation (e.g., Fp1 for eye blinks, jaw for mandibular muscle activity). This not only facilitates signal cleaning but also allows for quantitative assessment of the relative importance of specific auxiliary electrodes in the artifact reduction process, which constitutes a central aspect of this study. Moreover, the LS method exhibits high stability and reproducibility, which is essential when analyzing data from multiple participants and recording sessions. Due to its deterministic formulation and the absence of stochastic optimization steps, it enables reliable inter-subject comparisons and systematic investigation of regression coefficient variability across channels and time. Finally, the regression coefficients ***β*** can be exploited not only for signal cleaning but also as a criterion for selecting auxiliary channels with the greatest physiological relevance. Mean absolute values |***β***| computed for each auxiliary channel provide a measure of its relative influence on the EEG signal, enabling identification of regressor sets that are optimal for individual participants. This approach combines the simplicity of classical linear regression with the ability to explore the spatial structure of EEG artifacts, offering both practical and interpretative benefits for the design of robust SSVEP-based BCI systems. The EEG signals were segmented into non-overlapping one-second time windows, which served as the basic units of analysis in the regression-based signal cleaning procedure. Time windows that are too short do not allow for reliable capture of more slowly varying artifact components, such as muscle- or motion-related disturbances, leading to unstable estimation of regression coefficients. Conversely, the use of longer windows would result in the aggregation of multiple artifacts with different temporal characteristics within a single regression model, violating the assumption of local stationarity and reducing artifact removal effectiveness. A one-second window provides a compromise between the temporal locality of the model and the stability of least-squares estimation, enabling effective modeling and reduction in artifacts while preserving the structure of the SSVEP signal. Figure 5 presents an example of a 5-s segment of signals recorded from the occipital electrodes O1, O2, and Oz, denoted as O-only (before signal cleaning), obtained from participant S01 during 9 Hz visual stimulation. Artifact occurrences are marked in red. Figure 6 shows an example of a 5-s segment of signals recorded from the same occipital electrodes after artifact removal, denoted as O-clean, from participant S01 during 9 Hz visual stimulation. Signal cleaning was performed using auxiliary channels, which enabled a substantial reduction in the influence of artifacts present in the original recordings. Figure 7 presents an example of a 1-s EEG window recorded from the Oz electrode in participant S03 during 9 Hz visual stimulation, illustrating the effect of artifact removal on both the time-domain waveform and the frequency-domain representation of the signal. The upper panels show the time-domain signal before cleaning (O-only) and after cleaning using least squares regression (O-clean). A clear reduction in signal amplitude is observed after cleaning, indicating effective attenuation of artifact-related components. The lower panels present the corresponding FFT amplitude spectra. Before cleaning, numerous spectral components with elevated amplitudes are visible, reflecting the presence of artifacts. After applying the cleaning procedure, the overall amplitude is substantially reduced, confirming effective suppression of artifacts while preserving the relevant EEG frequency components. Notably, the 9 Hz component associated with the visual stimulation becomes more prominent after cleaning, demonstrating improved signal clarity.

### 5.2. Extraction of Informative Features from EEG Signals for SSVEP Detection

To obtain features suitable for SSVEP response classification, frequency-domain analysis using the fast Fourier transform (FFT) was performed for both analysis variants: O-only (raw signals from the occipital electrodes O1, O2, Oz) and O-clean (signals cleaned using least squares linear regression with auxiliary channels) [27,55,56]. The analysis was carried out on 1-s windows (*N* = 256 samples, *fs* = 256 Hz), without overlap, providing a frequency resolution of 1 Hz while maintaining sufficient temporal sensitivity. For feature extraction, the EEG signals were segmented into non-overlapping one-second time windows. This choice provides a frequency resolution of 1 Hz, which is sufficient to unambiguously distinguish the SSVEP stimulus frequencies (7, 8, and 9 Hz) and their harmonics in the spectral domain. The use of shorter windows would degrade frequency resolution, leading to spectral leakage and overlap of adjacent frequency bins, thereby hindering precise identification of the stimulus-related components. Conversely, longer windows would reduce the temporal locality of the extracted features and could mask amplitude variations in the SSVEP response resulting from the nonstationary nature of the EEG signal. A one-second window therefore represents a compromise between frequency resolution and preservation of the local temporal characteristics of the SSVEP response.

For each window, the FFT amplitude spectrum in the 0–30 Hz range was computed, and amplitudes corresponding to the visual stimulation frequencies and their harmonics were extracted. Three stimulation frequencies were used in the study: 7 Hz, 8 Hz, and 9 Hz. For each frequency, three harmonics—*f*, 2*f*, and 3*f*—were analyzed, corresponding to the spectral components at [7, 8, 9, 14, 16, 18, 21, 24, 27 Hz]. For each occipital channel (O1, O2, Oz), FFT amplitudes at the selected frequencies were computed and concatenated to form a feature vector representing a single analysis window [57,58]. The O-only variant reflected the direct EEG recordings, whereas the O-clean variant represented signals after subtraction of the artifact component estimated from the auxiliary channels. This design enabled a direct comparison of classification performance between raw and cleaned signals. Feature extraction in the frequency domain provides representations with clear physiological relevance, as the amplitudes of selected FFT components reflect the strength of neuronal synchronization with the visual stimulus. Moreover, the use of a simple and interpretable feature model (FFT with analysis of three harmonics) facilitated straightforward integration with SVM and CNN classifiers and allowed unambiguous interpretation of differences between data variants.

### 5.3. EEG Signal Classification

To assess the impact of LS linear regression on SSVEP recognition performance, two classification models were employed: a support vector machine (SVM) with a linear kernel and a convolutional neural network (CNN) operating on feature vectors [27,59,60,61]. Both models used the same frequency-domain representation based on FFT amplitudes. Classification was performed in parallel for two variants of the occipital signal:

- O-only: raw signals from the O1, O2, and Oz channels;
- O-clean: signals from the O1, O2, and Oz channels after LS regression with respect to an optimally selected subset of auxiliary channels (Cz, Fp1, HEOG, neck, cheek, jaw).

For each analysis window, amplitudes from the selected spectral components and all three occipital channels were concatenated into a single feature vector. In both variants (O-only and O-clean), the feature definition was identical. Only the input signal differed (raw vs. LS-regressed). Consequently, performance differences between variants reflect exclusively the effect of artifact reduction rather than changes in feature representation.

For SSVEP classification, a multi-class SVM with a linear kernel was used [60]. The model was trained using FFT-based features derived from either the O-only or O-clean variant, depending on the analyzed configuration. Each model was trained on a designated training set within a given data split, while performance evaluation was conducted on an independent test set. The choice of a linear kernel is consistent with the assumption that the amplitudes of spectral components corresponding to stimulation frequencies and their harmonics lead to largely linear separability of the 7 Hz, 8 Hz, and 9 Hz classes in the feature space. This model design simplifies result interpretation, reduces the number of parameters, and lowers the risk of overfitting, given the limited number of training examples. The SVM therefore serves as a classical, well-established baseline against which the neural network model can be compared.

The second model was based on a convolutional neural network trained directly on the FFT-derived feature vectors [61], identical to those used for the SVM classifier. Given the limited dataset size and the relatively low complexity of the task (three classes corresponding to 7 Hz, 8 Hz, and 9 Hz), a compact network architecture with a small number of parameters was adopted. This design choice reduces the risk of overfitting while maintaining adequate generalization capability. The network consists of an input normalization layer, a single fully connected layer with ReLU activation, a dropout layer for regularization, and a final softmax classification layer. The selection of such a simple architecture reflects the fact that the input data (FFT feature vectors) already constitute a high-level representation of the signal, making deep convolutional structures for spatial or temporal pattern extraction unnecessary. The detailed architecture and parameter settings of the convolutional neural network are summarized in Table 1. Training was performed using the Adam optimizer for up to 40 epochs with a batch size of 64. In each leave-one-subject-out (LOSO) split [55], an internal validation set (approximately 12.5% of the training samples, with class balance preserved) was extracted to monitor the training process. An early stopping mechanism with a patience of 3 epochs was applied.

To reduce the impact of random weight initialization, the network was trained three times for each variant (O-only and O-clean). The reported classification accuracy corresponds to the mean of the three runs, together with the associated statistical variability (standard deviation, SD). The CNN serves as a nonlinear classifier operating in the same harmonic feature space as the SVM, enabling evaluation of whether additional nonlinear modeling provides benefits once the occipital signal has been preprocessed using LS regression.

A leave-one-subject-out (LOSO) evaluation strategy was adopted: in each iteration, all windows from one participant formed the test set, while data from the remaining participants constituted the training set [62,63]. This approach allows assessment of system performance on previously unseen users who were not included in the training phase. The primary performance metric was classification accuracy, defined as the percentage of correctly classified 1-s windows in the test set. For a three-class SSVEP problem, this measure has direct practical relevance, as it corresponds to the probability of correctly decoding the user’s intent in a single BCI decision step. Comparing accuracy between the O-only and O-clean variants, while keeping the FFT feature representation and the SVM/CNN models unchanged, allows the observed performance gains to be attributed unambiguously to the artifact reduction process based on LS linear regression.

## 6. Results and Discussion

### 6.1. Comparison of Classification Accuracy After Artifact Removal Using Auxiliary Electrodes

The study aimed to evaluate the effectiveness of an EEG artifact cleaning method in the context of SSVEP classification using SVM and CNN models. Two data variants were analyzed: raw signals from occipital electrodes (O-only) and signals cleaned using linear regression with auxiliary channels (O-clean). A leave-one-subject-out (LOSO) cross-validation procedure was employed to assess model generalization across participants. For both classification approaches, a clear improvement in classification accuracy was observed after applying signal cleaning. Analysis of SSVEP classification results obtained using the SVM (Table 2) and CNN (Table 3) models demonstrated a clear and consistent impact of the proposed EEG artifact cleaning method (O-clean variant) on recognition performance.

For the SVM classifier, the mean classification accuracy increased from 70.8 ± 20% to 79.9 ± 17.3%, corresponding to an average improvement of +9.1 ± 6.4 percentage points. A comparable effect was observed for the CNN model, where mean accuracy improved from 70.7 ± 21.1% to 79.7 ± 18.5%, yielding an average gain of +9.9 ± 8.2 pp. The largest individual improvements in classification accuracy were observed for participants S1 (+25.6 pp for SVM and +29.4 pp for CNN) and S11 (+16.7 pp and +22.4 pp, respectively), indicating that the regression-based method is particularly beneficial for signals affected by strong muscular and motion-related artifacts. In the vast majority of cases, performance gains were positive. Only a single participant (S7, CNN) exhibited a marginal decrease (−1.0 pp), confirming the overall stability and robustness of the proposed approach.

The statistical significance of the performance improvement was assessed at the level of individual subjects using McNemar’s test, which compares classifier decisions for the same test windows. For the SVM method, a statistically significant improvement in classification accuracy (*p* < 0.05) was obtained for the following subjects: S1, S3, S4, S5, S8, S10, S11, and S12. For these users, the number of windows correctly classified exclusively in the O-clean variant was significantly greater than the number correctly classified exclusively in the O-only variant, indicating a systematic improvement in classifier decisions after regression-based signal cleaning. For subjects S2, S6, and S9, only a slight increase in mean accuracy was observed, which did not reach statistical significance. In these cases, the number of improved and degraded windows was comparable, suggesting that the O-clean variant did not lead to a clear improvement in classification quality. For subject S7, the increase in mean accuracy was limited, and McNemar’s test did not reveal a significant difference between the variants, indicating no benefit from regression-based signal cleaning in this specific case.

An analogous statistical significance analysis was performed for the CNN classifier, also using McNemar’s test at the level of individual test windows. A statistically significant improvement in classification accuracy (*p* < 0.05) was observed for subjects S1, S3, S4, S5, S9, S10, S11, and S12. For these users, the O-clean variant led to a significant increase in the number of correct window-level decisions compared with the O-only variant. For subjects S2, S6, and S8, the improvement in mean accuracy was not statistically significant. In particular, for subject S6, a high variability in CNN decisions at the window level was observed, resulting in a similar number of improved and degraded classifications after signal cleaning. For subject S7, not only was no significant improvement obtained, but no consistent effect of signal cleaning was observed, as reflected by the non-significant McNemar’s test result and the minimal change in classification accuracy.

The improvement in classification performance obtained with the O-clean variant is statistically significant at the population level (*p* < 0.001). This indicates that the observed increase in mean accuracy is not driven by isolated cases but represents a systematic effect across the entire group. Similarly, for the CNN classifier, the application of the O-clean variant results in a statistically significant improvement in classification accuracy at the subject level (*p* < 0.001). Despite greater variability in individual performance gains, the effect of signal cleaning remains consistent across the studied population.

The practical relevance of the observed average accuracy increase of approximately 9–10 percentage points depends on the characteristics of each subject’s EEG signal. In cases where baseline performance was moderate or low (around 40–60%), such as for subjects S1, S3, S8, and S11, improvements on the order of several tens of percentage points represent a qualitative change in system performance and may determine its practical usability. In contrast, for subjects with high initial accuracy (above 90%), such as S2, S4, and S12, gains of 2–4 percentage points have limited functional significance, as the classifier was already operating close to its maximum prior to signal cleaning. Consequently, the average improvement (~9–10 pp) reflects the fact that regression-based signal cleaning is particularly beneficial for users with a higher proportion of artifacts and lower EEG signal quality, while its impact diminishes as baseline signal quality increases.

**Figure 8:**
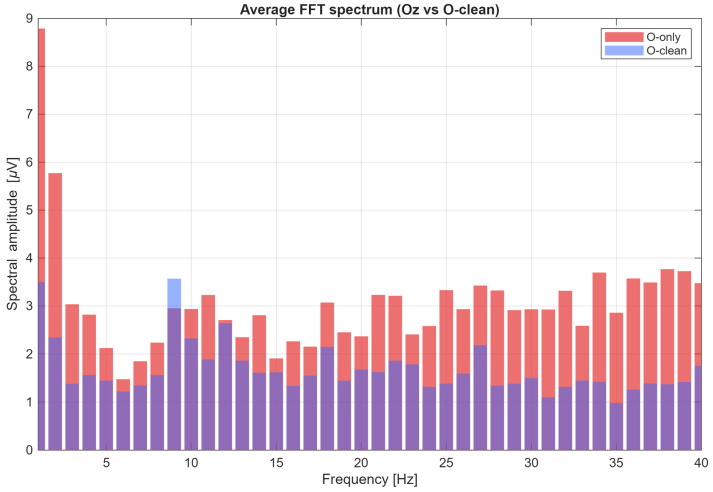
Averaged FFT amplitude spectrum (1–40 Hz) of the EEG signal from the Oz electrode during 9 Hz visual stimulation, before cleaning (O-only) and after artifact removal (O-clean).

Figure 8 presents the averaged FFT amplitude spectrum of the EEG signal from the Oz electrode in the 1–40 Hz range during 9 Hz visual stimulation, comparing signals before artifact removal (O-only) and after cleaning (O-clean). The spectrum prior to cleaning exhibits elevated amplitude levels across a broad frequency range, reflecting substantial contamination by artifact-related components. After applying the cleaning procedure, a pronounced reduction in mean amplitude is observed throughout the analyzed band, while the spectral component corresponding to the 9 Hz stimulation frequency remains clearly preserved. This result confirms the effectiveness of the proposed method in suppressing artifacts while retaining information that is essential for SSVEP analysis. The application of linear regression with auxiliary channels enabled effective reduction in non-physiological interference, resulting in improved EEG signal quality and increased reliability of SSVEP classification. The obtained results clearly confirm the validity of signal cleaning in BCI systems, as it led to an average accuracy improvement of approximately 9–10 percentage points, which can substantially enhance the practical reliability of brain–computer interfaces.

Table 4 presents the Information Transfer Rate (ITR) [64], expressed in bits per minute, computed for two SSVEP classification models (SVM and CNN) assuming three stimulus classes and 1-s analysis windows.

The ITR values were calculated based on the mean classification accuracies obtained using the leave-one-subject-out evaluation procedure. The application of linear regression with auxiliary channels (O-clean variant) resulted in a significant increase in both classification accuracy and information transfer rate (ITR). For the SVM classifier, the mean ITR increased by approximately 16.5 bit/min, while for the CNN model, the improvement reached 13.9 bit/min. This corresponds to an enhancement in communication efficiency of approximately 60% compared to the raw signal. These results confirm that EEG artifact removal substantially improves the practical efficiency and reliability of SSVEP classification and, consequently, the overall performance of BCI systems.

### 6.2. Comparison of Classification Accuracy Using CCA and FBCCA Before and After Artifact Reduction

Two classical reference methods for SSVEP classification were employed in the analysis: Canonical Correlation Analysis (CCA) and Filter Bank Canonical Correlation Analysis (FBCCA). The analysis was performed on non-overlapping one-second EEG signal windows (256 samples at a sampling frequency of 256 Hz), in the same manner as for the previously applied SVM and CNN classifiers, which allowed for a direct comparison of results.

In the CCA method, a set of reference signals was generated for each stimulus frequency (7, 8, and 9 Hz) in the form of sine and cosine functions, including the first three harmonics of the fundamental frequency. Thus, for each class, a reference matrix containing sinusoidal components at frequencies *f*, 2*f*, and 3*f* was constructed. Subsequently, for each EEG window, canonical correlation coefficients were computed between the EEG signal and each reference matrix. Classification was performed by selecting the stimulus frequency for which the largest value of the first canonical correlation coefficient was obtained. This method is robust to unknown phase shifts between the stimulus and the EEG response, as it simultaneously exploits sine and cosine components.

In the FBCCA method, the classification procedure extended the classical CCA by incorporating a bank of band-pass filters. Five fourth-order Butterworth filters, implemented in a zero-phase manner, were used with the following frequency ranges: 6–90 Hz, 14–90 Hz, 22–90 Hz, 30–90 Hz, and 38–90 Hz. Each EEG window was filtered independently in each band, and CCA-based classification was then performed for each filtered signal using the same set of reference signals. As a result, for each stimulus frequency, five canonical correlation values corresponding to the individual filter bands were obtained.

Table 5 presents the classification accuracies achieved with the CCA method in the three-class SSVEP recognition task, for both the O-only and O-clean signal variants, evaluated using the leave-one-subject-out scheme. The final classification decision in FBCCA was based on a weighted sum of the canonical correlation coefficients obtained across the individual frequency bands. The weights assigned to successive bands decreased with increasing lower cutoff frequency, reflecting the fact that lower SSVEP harmonics typically carry more information than higher-order harmonics. Consequently, a single decision value was computed for each stimulus frequency, and classification was performed by selecting the frequency with the highest value. Table 6 reports the classification accuracies for the FBCCA method in the same three-class SSVEP task, also for the O-only and O-clean signal variants, and evaluated using the leave-one-subject-out scheme. Classification performance was evaluated using the Leave-One-Subject-Out protocol, enabling an assessment of the methods’ ability to generalize to previously unseen users.

The results presented in the tables indicate that regression-based artifact reduction (the O-clean variant) leads to a systematic improvement in SSVEP classification performance for both CCA and FBCCA under the leave-one-subject-out evaluation scheme. For classical CCA, the mean accuracy increased from 73.6% for the raw signal (O-only) to 82.0% after signal cleaning, corresponding to an average improvement of 7.4 percentage points. The largest gains were observed for subjects with low baseline performance, where improvements reached up to 20 percentage points, whereas for participants with high signal quality, the changes were small or negligible.

A similar trend was observed for the FBCCA method, for which the mean accuracy increased from 73.9% in the O-only variant to 80.1% in the O-clean variant, corresponding to an improvement of 6.2 percentage points. This improvement was less pronounced than for CCA, suggesting that the filter bank partially compensates for the presence of noise already at the band-pass filtering stage. In individual cases, slight decreases in accuracy were observed after signal cleaning, indicating subject-specific differences in artifact structure and in their correlation with the auxiliary channels.

Overall, the results confirm that regression-based artifact reduction improves the stability of canonical correlations and enhances the ability of both CCA and FBCCA methods to generalize to new users, with a more pronounced effect observed for classical CCA than for its filter-bank-based variant.

### 6.3. Analysis of the Signal-to-Noise Ratio of SSVEP Signals After Regression-Based EEG Cleaning

One of the key factors determining the effectiveness of EEG signal analysis and classification methods is the signal-to-noise ratio (SNR). In the case of steady-state visually evoked potentials, signal quality is particularly limited by the presence of non-neuronal disturbances, such as ocular artifacts, muscle-related components, and slow potential fluctuations. For this reason, in addition to evaluating classification performance, it is important to directly quantify the impact of the applied signal-cleaning methods on the spectral properties of the EEG.

In this analysis, the effect of EEG signal cleaning on the SNR of SSVEP responses recorded from the occipital electrodes O1, O2, and Oz was evaluated. The analysis was conducted for three visual stimulus frequencies: 7 Hz, 8 Hz, and 9 Hz. The computations were performed separately for each subject, and the results were subsequently averaged to obtain group-level measures. The EEG signals were segmented into one-second windows and transformed into the frequency domain using the discrete Fourier transform. The SNR for a stimulus frequency *f* was calculated as the logarithmic ratio of the spectral power of the signal at the frequency bin corresponding to the stimulation frequency to the mean spectral power in neighboring frequency bins, representing the background EEG activity. Formally, the SNR was expressed in decibels according to the following relation:

(4) $$\mathrm{SNR}\left(f\right)=10\log_{10}\left(\frac{P\left(f\right)}{\frac{1}{K}\sum_{f^{\prime }\in N\left(f\right)}P\left(f^{\prime }\right)}\right)$$

where *P*(*f*) denotes the spectral power of the EEG signal in the frequency bin corresponding to the stimulation frequency *f*, and *P*(*f*′) refers to the spectral power in the neighboring frequency bins representing the EEG background. The set of background bins comprised six adjacent frequencies (*f* − 3 Hz, *f* − 2 Hz, *f* − 1 Hz, *f* + 1 Hz, *f* + 2 Hz, *f* + 3 Hz), excluding the signal bin itself, corresponding to *K* = 6. SNR values were computed separately for each window and subsequently averaged within each subject and stimulation frequency. The O-only variant corresponded to unprocessed signals, whereas the O-clean variant included signals after regression-based removal of interfering components using additional reference channels. The values reported in Table 7 represent mean signal-to-noise ratios calculated for the occipital electrodes O1, O2, and Oz and then spatially averaged for each subject and stimulus frequency. This approach reduces the influence of local amplitude differences across occipital electrodes and provides a more stable and representative assessment of SSVEP response quality.

For all analyzed stimulus frequencies (7, 8, and 9 Hz), a systematic increase in SNR was observed after applying regression-based EEG signal cleaning. The largest mean improvement was obtained for the 7 Hz stimulus (+1.7 dB), while slightly smaller but comparable SNR gains were observed for 8 Hz (+1.6 dB) and 9 Hz (+1.3 dB). These results indicate that the removal of artifact-related components effectively enhances the spectral components associated with the SSVEP response, regardless of the stimulation frequency. The obtained results confirm that regression-based signal cleaning improves the spectral quality of SSVEP signals. The increase in SNR provides a direct explanation for the previously observed improvements in classification performance, indicating that they arise from a genuine enhancement of the contrast between stimulus-related activity and background noise, rather than merely from the operation of the classification algorithms.

### 6.4. Significance and Selection of Auxiliary Channels in the EEG Artifact Cleaning Process

To determine which auxiliary channels contribute most to the EEG artifact reduction process, regressor selection was performed within the linear regression (LS) model. For each participant, all possible combinations of auxiliary channels were analyzed, including central (Cz), frontal (Fp1), oculomotor (HEOG), and muscular electrodes (neck, cheek, jaw), yielding a total of 63 combinations. For each configuration, cleaned signals (O-clean) were computed for the occipital channels O1, O2, and Oz, and the effectiveness of SSVEP classification after artifact reduction was subsequently evaluated. For each participant, the set of regressors that provided the highest post-cleaning classification accuracy was selected. The results obtained for the SVM and CNN models were fully consistent, allowing them to be consolidated into a single table presenting the optimal auxiliary channel sets. It is worth emphasizing that the best classification performance was achieved for variants employing regressor sets based on auxiliary electrode signals, confirming their crucial role in the physical modeling and elimination of EEG artifacts. Table 8 presents the best auxiliary channel combinations selected for each participant.

The results presented in Table 8 indicate that the Cz electrode was included in every optimal regressor set, confirming its dominant role. The Cz signal provided a stable reference for artifact components common across the EEG montage and enabled effective compensation of muscle- and posture-related fluctuations. The Fp1 and HEOG channels frequently appeared as complements to Cz, highlighting their importance in reducing ocular- and frontal-origin artifacts. In addition, the muscular channels of the jaw and cheek proved relevant in cases where interference was induced by jaw muscle tension and facial expressions.

The most frequently selected auxiliary channels were: Cz in 12/12 participants, Fp1 in 6/12, HEOG in 6/12, jaw in 4/12, and cheek in 4/12 participants. The universal inclusion of the Cz electrode suggests that signals from central scalp regions capture global interference components and serve as the most robust and versatile regressor for EEG artifact cleaning models. The complementary roles of Fp1 and HEOG are particularly important for compensating eye movements and frontal muscle activity, while jaw and cheek channels contribute information related to lower facial muscle artifacts. These conclusions indicate that effective artifact suppression in EEG–BCI systems should include at least one central channel (Cz) together with electrodes capturing muscular and oculomotor activity. Such a configuration enables a substantial improvement in signal quality and enhances classification accuracy under real-world operating conditions.

### 6.5. Analysis of Regression Coefficient Values for Auxiliary Channels

To quantitatively assess the role of auxiliary channels in cleaning occipital EEG signals (O1, O2, Oz), the values of linear regression coefficients (***β***) obtained from the least squares (LS) model were analyzed for electrodes. For each 1-s window, a ***β*** vector was estimated; subsequently, results were aggregated across all windows and participants to compute the mean absolute regression coefficient (|***β***|), the standard deviation (SD), and the coefficient of variation (CV = SD/|***β***|), which serves as a measure of the relative variability of each channel. A low CV indicates a stable regressor influence, whereas high values reflect strongly time-varying behavior.

The analysis revealed that the Cz electrode played a dominant role in the LS regression model, exhibiting the highest mean |***β***| and relatively low variability (CV ≈ 0.42). This indicates that signals from central scalp regions consistently capture global interference components also present in occipital channels, making Cz the most reliable regressor for artifact compensation. The Fp1 and HEOG channels showed lower mean |***β***| values, yet their contribution remained substantial, confirming their importance in reducing ocular and frontal artifacts. The high coefficients of variation (CV > 1) for these channels suggest an episodic nature, strongly dependent on eye movements and frontal muscle activity. The muscular channels Cheek and Jaw exhibited pronounced |***β***| values combined with the highest variability (CV ≈ 1.3–1.5), reflecting the dynamic and individual-specific nature of facial and mandibular artifacts that occur irregularly during visual stimulation. The Neck channel yielded the lowest mean |***β***| values but a moderate coefficient of variation, indicating a relatively consistent influence of neck muscle tension associated with head posture maintenance.

In summary, the results clearly confirm that the Cz channel serves as the primary and stable regressor in the EEG cleaning model, while Fp1 and HEOG play key roles in compensating ocular and frontal artifacts. The muscular channels Cheek and Jaw complement the model by providing information about dynamic muscle-related interference. An optimal regressor set should therefore combine a central electrode with channels capturing muscular and oculomotor activity, enabling more effective artifact reduction and improved SSVEP classification accuracy under conditions representative of real-world BCI applications. Table 9 presents the mean LS regression coefficients (|***β***|), standard deviations (SD), and coefficients of variation (CV) for auxiliary channels. Table 10 presents the mean absolute values of the linear regression coefficients (|***β***|) for auxiliary channels, computed separately for each participant (S1–S12). These values reflect the relative contribution of individual channels to the LS regression model and indicate which electrodes exerted the greatest influence on artifact removal in occipital EEG signals.

Analysis of the obtained values indicates that the largest mean regression coefficients (|***β***|) are associated with the Cz electrode, confirming its dominant role in the LS regression model. Signals from central scalp regions capture global interference components common across the EEG montage and thus constitute the most stable regressor for artifact compensation. The Fp1 and HEOG channels also exhibit a substantial contribution to the model, highlighting their importance in reducing ocular and frontal artifacts such as blinking and eye movements. The muscular channels of the cheek and jaw are characterized by moderate |***β***| values but high inter-participant variability, reflecting individual differences in facial and mandibular muscle activity during recording. In contrast, the neck channel shows a more consistent influence, corresponding to stable neck muscle tension associated with head posture. Overall, these results clearly demonstrate that an optimal set of auxiliary channels in EEG cleaning models should include at least one central electrode (Cz) together with channels capturing muscular and oculomotor activity. Such a configuration enables effective artifact suppression and improves the accuracy of SSVEP signal classification in BCI applications, particularly under naturalistic conditions where artifacts are unavoidable.

### 6.6. Comparison of the Obtained Results with the Literature and Implications for Future Research

The results presented in this study confirm that muscular, motion-related, and ocular artifacts constitute one of the principal factors limiting the effectiveness of SSVEP classification, and that their removal leads to measurable improvements in BCI system performance. The observed average increase in classification accuracy of approximately 9–10 percentage points for both applied classifiers (SVM and CNN), together with an increase in ITR of 13.9–16.5 bit/min for a 1-s decision window, is consistent with trends reported in recent studies on the robustness of SSVEP to physiological disturbances.

The literature has demonstrated that EMG and motion artifacts lead to a significant attenuation of SSVEP components and their harmonics, which directly degrades the separability of spectral features used for classification. In Ref. [48], the authors showed that neck and jaw muscle activity, facial expressions, and actions such as swallowing cause a pronounced reduction in signal power within the stimulation frequency band, resulting in decreased SSVEP recognition performance even when classical spectral analysis methods are employed. Similar conclusions have been reported in studies examining the impact of jaw muscle tension on SSVEP responses, where it was shown that even brief jaw clenching leads to substantial distortions in signals recorded from occipital electrodes and a reduction in stimulus-frequency classification accuracy. The authors emphasize that such artifacts are particularly problematic in practical applications, in which users are unable to maintain complete facial muscle relaxation over extended periods.

With respect to artifact reduction methods, comparative studies indicate that the effectiveness of signal cleaning should be evaluated primarily in terms of its impact on final SSVEP classification performance, rather than solely on signal-quality measures in the time or frequency domain. Jurczak et al. [65] compared ICA, linear regression, and adaptive filtering in SSVEP tasks affected by muscle and motion artifacts, showing that improvements in classification accuracy following artifact reduction typically range from several to over a dozen percentage points, depending on the type of disturbance and the method applied. An important conclusion of this work is that regression-based and adaptive methods—despite their lower complexity—provide stable performance gains without the risk of excessive attenuation of neural components. More broadly, reviews of EEG processing methods emphasize that optimizing input signal quality often yields greater benefits than further increasing classifier complexity. Article [5] highlights that EMG and EOG artifacts remain among the main factors limiting BCI performance, and that artifact reduction methods are particularly critical in low-channel systems and mobile applications.

Against this background, the results obtained in the present study have clear practical significance. The magnitude of improvement in classification accuracy and ITR is comparable to that achieved by more complex methods, while maintaining substantially lower computational complexity. Moreover, analysis of the regression coefficients enabled unambiguous identification of auxiliary electrodes with the greatest relevance for artifact modeling—most notably the central Cz channel and electrodes associated with facial muscle activity and eye movements. Such quantitative and physiologically interpretable insights are rarely available in ICA-based or deep-learning approaches.

A comparison of the obtained results with the existing literature suggests several natural directions for future research. First, it appears justified to extend the proposed regression-based approach toward adaptive methods, such as the recursive least squares (RLS) algorithm. In the present study, regression parameters are estimated in a windowed manner, which allows for partial tracking of slowly varying artifacts. However, the application of fully adaptive algorithms could further improve robustness to nonstationary artifacts occurring during longer recording sessions or under changing experimental conditions. It should be emphasized that increased adaptivity entails a higher computational cost, indicating a trade-off between signal-cleaning accuracy and computational complexity, which should be systematically investigated in future work. Second, identification of a minimal electrode set (Cz and selected EMG/EOG channels) provides a foundation for designing low-channel, wearable SSVEP–BCI systems in which artifact reduction is addressed already at the signal acquisition stage. Finally, future studies may combine simple and interpretable signal-cleaning methods with more advanced SSVEP detection algorithms, while preserving the central role of input signal quality as the key determinant of overall system performance.

## 7. Conclusions

This work proposes and validates an EEG artifact reduction method for SSVEP–BCI systems based on linear regression using auxiliary channels that include central, frontal, oculographic, and muscular electrodes. The obtained results clearly confirm that muscle, motion-related, and ocular artifacts significantly limit the effectiveness of SSVEP classification, and that their explicit modeling and removal lead to substantial improvements in signal quality and overall BCI system performance. Applying regression in 1-s time windows resulted in an average increase in classification accuracy of approximately 9–10 percentage points for both a linear SVM classifier and a compact CNN, accompanied by an increase in information transfer rate (ITR) of 13.9–16.5 bit/min. Notably, these improvements were achieved without modifying the feature definition or the complexity of the classification models, which unequivocally indicates that the primary factor driving the performance gain was the enhancement of input signal quality.

Analysis of regressor selection revealed the dominant role of the central electrode Cz, which was included in the optimal auxiliary channel sets for all participants. Frontal and oculographic channels (Fp1, HEOG) proved important for compensating ocular artifacts, while muscle electrodes located on the jaw and cheek effectively modeled dynamic EMG artifacts. Examination of the regression coefficients confirmed both the stable influence of the Cz channel and the episodic yet often strong contribution of muscular channels.

The results indicate that effective artifact reduction should be an integral component of signal processing in SSVEP–BCI systems, particularly under conditions approaching real-world use. The proposed approach is characterized by low computational complexity, high stability, and full physiological interpretability, making it an attractive solution for practical BCI applications, including low-channel and wearable systems.

## Figures and Tables

**Table 1:** Convolutional neural network architecture.

Stage	Layer	Parameter	Aim
Input	featureInputLayer(nFeat, ‘Normalization’, ‘zscore’)	nFeat = number of features (3 channels × 6 bands)	Normalization of FFT feature vectors to standardize the value ranges
Hidden layer	fullyConnectedLayer(64) + reluLayer	64 neurons with ReLU activation	Nonlinear feature transformation and extraction of inter-feature dependencies
Regularization	dropoutLayer(0.5)	Dropout probability = 0.5	Overfitting reduction through random neuron deactivation (dropout)
Output	fullyConnectedLayer(3)	Three output neurons (classes 7, 8, 9 Hz)	Mapping feature representations to the class space
Classification	softmaxLayer, classificationLayer	Normalization of probabilities, cost function	Computation of class probability distributions and final classification decision

**Table 2:** Classification accuracy (%) for the SVM method in the three-class SSVEP recognition task, with classifier training and testing performed using the leave-one-subject-out scheme.

Subject	O-Only	O-Clean	Improvement
S1	52.2	77.8	+25.6
S2	90.0	93.3	+3.3
S3	55.6	68.5	+12.9
S4	91.1	95.6	+4.5
S5	92.8	99.4	+6.6
S6	90.4	94.3	+3.9
S7	53.3	59.4	+6.1
S8	41.1	48.9	+7.8
S9	52.2	59.4	+7.2
S10	81.1	92.2	+11.1
S11	53.3	70.0	+16.7
S12	96.1	99.4	+3.3
Mean	70.8 ± 20	79.9 ± 17.3	+9.1 ± 6.4

**Table 3:** Classification accuracy (%) for the CNN method in the three-class SSVEP recognition task, with classifier training and testing performed using the leave-one-subject-out scheme.

Subject	O-Only	O-Clean	Improvement
S1	48.7	78.1	+29.4
S2	90.6	93.3	+2.7
S3	57.2	66.9	+9.7
S4	92.1	96.0	+3.9
S5	89.3	99.4	+10.1
S6	93.1	94.4	+1.3
S7	53.0	52.0	−1.0
S8	40.0	48.0	+8.0
S9	49.6	58.7	+9.1
S10	87.4	95.7	+8.3
S11	51.5	73.9	+22.4
S12	95.6	99.4	+3.8
Mean	70.7 ± 21.1	79.7 ± 18.5	+9.9 ± 8.2

**Table 4:** Information transfer rate (ITR) values and corresponding ITR improvement, computed for 1-s windows assuming three classes (7, 8, and 9 Hz).

Model	Variant	ITR [bit/min]	Improvement
SVM	O-only	27.5	-
	O-clean	44.1	+16.5
CNN	O-only	25.2	-
	O-clean	39.1	+13.9

**Table 5:** Classification accuracy (%) for the CCA method in the three-class SSVEP recognition task, using O-only and O-clean signal variants, with classifier evaluation performed under the leave-one-subject-out scheme.

Subject	O-Only	O-Clean	Improvement
S1	52.8	72.8	+20.0
S2	96.7	96.7	+0.0
S3	59.4	69.4	+10.0
S4	97.8	97.8	+0.0
S5	81.1	95.6	+14.5
S6	71.7	86.1	+14.4
S7	85.0	86.1	+1.1
S8	87.8	92.2	+4.4
S9	48.9	47.8	−1.1
S10	55.6	73.9	+18.3
S11	53.3	55.0	+1.7
S12	92.8	98.3	+5.5
Mean	73.6 ± 17.9	81.0 ± 16.5	7.4 ± 7.4

**Table 6:** Classification accuracy (%) for the FBCCA method in the three-class SSVEP recognition task, using O-only and O-clean signal variants, with classifier evaluation performed under the leave-one-subject-out scheme.

Subject	O-Only	O-Clean	Improvement
S1	60.6	71.7	+11.1
S2	96.1	96.7	+0.6
S3	58.9	68.3	+9.4
S4	97.8	97.8	+0.0
S5	80.0	95.6	+15.6
S6	76.1	85.6	+9.5
S7	86.7	86.1	−0.6
S8	82.2	87.8	+5.6
S9	47.8	45.0	−2.8
S10	61.1	78.3	+17.2
S11	51.1	53.7	+2.6
S12	88.9	95.0	+6.1
Mean	73.9 ± 16.7	80.1 ± 16.6	+6.2 ± 6.2

**Table 7:** Signal-to-noise ratio before and after cleaning for target SSVEP frequencies.

Frequency [Hz]	SNR O-Only [dB]	SNR O-Clean [dB]	SNR Gain [dB]
7 Hz	3.3 ± 4.1	5.0 ± 4.5	+1.7 ± 1.2
8 Hz	4.1 ± 4.3	5.7 ± 4.6	+1.6 ± 1.0
9 Hz	4.5 ± 3.8	5.8 ± 4.1	+1.3 ± 1.1

**Table 8:** Best auxiliary channel combinations selected for each participant.

Subject	Most Effective Auxiliary Electrodes
S01	Cz + HEOG + cheek
S02	Cz + cheek
S03	Cz + HEOG + jaw
S04	Cz
S05	Cz + Fp1 + jaw
S06	Cz
S07	Cz
S08	Cz + Fp1
S09	Cz + Fp1 + HEOG
S10	Cz + HEOG + jaw
S11	Cz + cheek
S12	Cz + Fp1 + HEOG

**Table 9:** Mean LS regression coefficients (|***β***|), standard deviations (SD), and coefficients of variation (CV) for auxiliary channels.

Channel	Mean	SD	CV	Characteristics
Cz	0.416	0.175	0.42	Stable, dominant regressor capturing global EEG interference components
Fp1	0.115	0.144	1.25	Frontal–ocular–muscular artifacts with an episodic character
HEOG	0.136	0.162	1.19	Oculomotor artifacts with a pronounced but highly variable influence
Neck	0.097	0.079	0.82	Moderately stable neck muscle–related artifacts
Cheek	0.127	0.164	1.28	Facial expressions producing strongly time-varying muscular signals
Jaw	0.132	0.199	1.51	Jaw muscle tension exhibiting the highest temporal variability

**Table 10:** Mean LS regression coefficient values for auxiliary channels.

Subject	Cz	Fp1	HEOG	Neck	Cheek	Jaw
S01	0.518	0.074	0.082	0.165	0.109	0.04
S02	0.399	0.071	0.124	0.106	0.081	0.278
S03	0.398	0.073	0.126	0.062	0.09	0.114
S04	0.425	0.143	0.088	0.152	0.111	0.073
S05	0.291	0.139	0.136	0.139	0.153	0.163
S06	0.31	0.108	0.14	0.123	0.161	0.182
S07	0.379	0.067	0.1	0.112	0.142	0.115
S08	0.354	0.112	0.098	0.146	0.138	0.097
S09	0.426	0.118	0.12	0.092	0.136	0.089
S10	0.405	0.102	0.132	0.129	0.149	0.141
S11	0.386	0.083	0.096	0.108	0.13	0.127
S12	0.373	0.094	0.109	0.115	0.144	0.121

## Data Availability

A database of EEG and EMG SSVEP recordings for artifact analysis and removal. https://github.com/kolodzima/EEG_artefact_SSVEP_EMG_EOG (accessed on 29 December 2025).

## Abbreviations

The following abbreviations are used in this manuscript:

ASR	Artifact subspace reconstruction
BCI	Brain–computer interface
BSS	Blind source separation
CCA	Canonical correlation analysis
CNN	Convolutional neural network
CV	Coefficient of variation
EEG	Electroencephalography
EMG	Electromyography
EOG	Electrooculography
FBCCA	Filter bank canonical correlation analysis
FFT	Fast Fourier transform
FIR	Finite impulse response
HEOG	Horizontal electrooculography
ICA	Independent component analysis
ITR	Information transfer rate
LED	Light-emitting diode
LMS	Least mean squares
LOSO	Leave-one-subject-out
LS	Least squares
MSI	Multivariate synchronization index
PCA	Principal component analysis
RLS	Recursive least squares
ReLU	Rectified linear unit
SD	Standard deviation
SNR	Signal-to-noise ratio
SSVEP	Steady-state visual evoked potential
SVM	Support vector machine
TRCA	Task-related component analysis
